# Actigraphy as an objective intra-individual marker of activity patterns in acute-phase bipolar disorder: a case series

**DOI:** 10.1186/s40345-017-0115-3

**Published:** 2018-03-07

**Authors:** Karoline Krane-Gartiser, Andreas Asheim, Ole Bernt Fasmer, Gunnar Morken, Arne E. Vaaler, Jan Scott

**Affiliations:** 1 Department of Mental Health, NTNU, Norwegian University of Science and Technology, P.O. box 3250 Sluppen, 7006 Trondheim, Norway; 2 Department of Psychiatry, St. Olav’s University Hospital, Trondheim, Norway; 3 Department of Mathematical Sciences, NTNU, Norwegian University of Science and Technology, Trondheim, Norway; 4 Center for Health Care Improvement in Mid-Norway, St. Olav’s University Hospital, Trondheim, Norway; 5 Section for Psychiatry, Department of Clinical Medicine, Faculty of Medicine and Dentistry, University of Bergen, Bergen, Norway; 6 Division of Psychiatry, Haukeland University Hospital, Bergen, Norway; 7 Academic Psychiatry, Institute of Neuroscience, Newcastle University, Newcastle upon Tyne, UK

**Keywords:** Actigraphy, Bipolar disorder, Mania, Intra-individual, Variability, Non-linear dynamics, Biological variable, Objective markers, Personalized medicine

## Abstract

**Background:**

Actigraphy could be an objective alternative to clinical ratings of motor activity in bipolar disorder (BD), which is of importance now that increased activity and energy are added as cardinal symptoms of (hypo)mania in the DSM-5 and commonly used rating scales give inadequate information about motor symptoms. To date, most actigraphy studies have been conducted in groups and/or used mean activity levels as the variable of interest. The novelty of this case series is therefore to indicate the potential of actigraphy and non-parametric analysis as an objective and personalized marker of intra-individual activity patterns in different phases of BD. To our knowledge, this is the first case series that provides an objective assessment of non-linear dynamics in within-person activity patterns during acute BD episodes.

**Results:**

We report on three cases of bipolar I disorder with 24-h actigraphy recordings undertaken during the first few days of two or more separate admissions for an acute illness episode, including admissions for individuals in different phases of BD, or with different levels of severity in the same phase of illness. For each recording, we calculated mean activity levels over 24 h, but especially focused on key measures of variability and complexity in activity. Intra-individual activity patterns were found to be different according to phase of illness, but showed consistency within the same phase. With increasing psychotic symptoms, there was evidence of a lower overall level and greater irregularity in activity. As such, sample entropy (a measure of irregularity) may have particular utility in characterizing mania and psychotic symptoms, while assessment of the distribution of rest versus activity over 24 h may distinguish between phases of BD within an individual.

**Conclusions:**

This case series indicates that objective, intra-individual, real-time recordings of patterns of activity may have clinical impact as a valuable adjunct to clinical observation and symptom ratings. We suggest that actigraphy combined with detailed mathematical analysis provides a biological variable that could become an important tool for developing a personalized approach to diagnostics and treatment monitoring in BD.

**Electronic supplementary material:**

The online version of this article (10.1186/s40345-017-0115-3) contains supplementary material, which is available to authorized users.

## Background

Increased activity and energy are now included alongside mood changes as cardinal symptoms of (hypo)mania in the Diagnostic and Statistical Manual, 5th edition (DSM-5) (American Psychiatric Association 2013). A systematic review confirmed that empirical evidence supports this change in the ‘criterion A’ symptoms for (hypo)mania (Scott et al. 2016). However, it also highlighted that most instruments used to rate activity, energy and mood changes were not fit for this purpose in bipolar disorders (BD) (Scott et al. 2016). For example, an item response theory (IRT) analysis of the utility of the Young Mania Rating Scale and Montgomery–Asberg Depression Rating Scale indicated that both these widely used scales were poorly constructed and inefficient, (e.g., they contained several items that provided little or no psychometric information and only measured a narrow band of severity of symptoms) (Prisciandaro and Tolliver 2016). Given the renewed importance of accurately assessing activity patterns, it is pertinent to consider the role of intra-individual actigraphy as an adjunct to or an alternative rating of activity.

Actigraphy is a validated method for recording rest–activity patterns by non-invasive measurement of wrist movement (Ancoli-Israel et al. 2003) and has frequently been used for the objective measurement of sleep patterns, and to assess putative state or even trait markers of BD (Geoffroy et al. 2015; Ng et al. 2015). However, nearly all published studies have used between-group comparisons of rest–activity cycles recorded for about 1–14 days (usually BD cases versus controls), rather than examining repeated recordings of intra-individual activity over time (Scott et al. 2016; Gershon et al. 2015). There are a few exceptions, e.g., the earliest studies of actigraphy included repeated assessments of small samples of inpatients (Kupfer et al. 1974; Wehr et al. 1980; Wolff et al. 1985; Weiss et al. 1974), and a study of ten cases employed actigraphy to assess patient outcomes following lithium withdrawal (Klein et al. 1992). Nevertheless, the actiwatches available in the 1980s lacked sophistication and data analytic strategies may have been too simplistic. More recently, Salvatore et al. (2008) reported mean levels of activity and sleep in individuals in manic/mixed states and again after recovery. However, the study focused on group outcomes and crucially, mixed states and mania cases were grouped together, which recent research suggests may be inappropriate (Scott et al. 2017).

In summary, it is increasingly clear that simple measures, such as mean activity levels, do not capture the variability or complexity of activity patterns in BD sufficiently (Scott et al. 2016; Krane-Gartiser et al. 2014; De Crescenzo et al. 2016). To date, only one study has explored fluctuations in depressive symptoms by using within-person actigraphy alongside more sophisticated mathematical modeling, such as non-parametric analyses of activity patterns (Gershon et al. 2015). However, we could find no publications that specifically used non-linear dynamic analyses of within-person actigraphy recordings to determine the similarities or differences in intra-individual activity patterns during acute illness episodes. Given this omission from the literature, we report on three cases of BD-I that were hospitalized during an acute illness episode on two or more occasions. Intra-individual 24-h actigraphy provided objective recording of activity patterns for (a) different levels of symptom severity within the same illness episode, (b) relapses of the same phase of illness, or (c) relapses in different phases of BD.

## Methods

The actigraphy research program at St. Olav’s University Hospital in Trondheim, Norway, was approved by the Regional Ethical Committee of Central Norway (2011/137), and the study rationale, methodology and assessments used are detailed elsewhere (Scott et al. 2017; Krane-Gartiser et al. 2014, 2016).

Cases included in the current report were identified from individuals who gave written informed consent to participate in a series of case–control studies that involved 24-h actigraphy. Capacity to consent was evaluated by a clinical specialist in psychiatry or psychology, and patients who were judged unable to consent were not included. Individuals with a primary diagnosis of BD [according to a multi-disciplinary expert consensus that applied ICD-10 research diagnostic criteria (WHO 1993)] and actigraphy monitoring for at least two separate hospital admissions were selected. De-identified information regarding key aspects of the clinical history, psychiatric assessments and medical treatment during the recording periods were obtained from electronic medical case records, and sleep–wake cycle data were obtained from actigraphy recordings undertaken for 24 h via a wrist-worn actiwatch (Actiwatch Spectrum, Philips Respironics Inc., Murrysville PA, USA).

### Activity measurements

Activity counts were recorded for 1-min intervals, and data were analyzed for the total recording time of 24 h. We calculated the following variables:

1. Mean activity level as counts per minute.
2. *Variability in activity* Reported as (a) the standard deviation in percent of mean activity (SD) as a measure of the intra-individual fluctuations from the mean, which is equivalent to the coefficient of variation; (b) the root mean squared successive difference (RMSSD) as a measure of the variability from minute to minute; (c) the RMSSD/SD ratio, which assesses the relationship between successive count variability and overall variability; and (d) a new estimate of activity level, namely the ratio between rest and activity which displays the temporal development of changes in activity levels over 24 h. Such a measure of activity is interesting to contrast with other measures that primarily track variability and regularity. The estimate is computed as the ratio of actigraphy samples being zero to non-zero within a time interval. This ratio can vary between 0, no movement, and 1, continuous movement. By applying a shifting interval, with some smoothing (Ramsay and Silverman 2005), a continuous measure is obtained.
3. *Complexity* Sample entropy as a measure of pattern complexity or degree of regularity of the time series. This non-linear dynamical method examines even short time series for similar sequences at a certain temporal distance and calculates the probability that two sequences are self-similar. A high sample entropy value indicates less self-similarity or increased disorder (Richman and Moorman 2000), and such pattern irregularity has been found in several activated psychiatric conditions, e.g., activated depression, mania and schizophrenia (Krane-Gartiser et al. 2014, 2016; Hauge et al. 2011). Since extended periods of rest will yield low entropy, we plotted the rest/activity ratio alongside sample entropy curves to allow comparison of measures.

## Results

Three cases had a diagnosis of BD-I and at least two actigraphy recordings and could be included. Details of clinical history and presentation are deliberately brief to maintain anonymity, but a summary of key clinical and actigraphy parameters are provided in Additional file 1: Tables S1–S3.

### Case 1—repeated admissions during an episode of mania with psychosis (post-partum onset)

Case 1 was aged ≤ 30 years and had a history of hospitalization for mania 10 years prior to the current presentation. She had been well during her pregnancy (her first), but she was admitted within 1 week post-partum with mania and psychotic symptoms. The first actigraphy recording was on day 6 of the admission and the daily medication included olanzapine 5 mg and oxazepam 20 mg. The admission lasted for about 2 weeks.

Readmission occurred about 1 month later because of a severe relapse of the manic episode with more marked psychotic symptoms. After readmission, the daily medication changed to quetiapine 250 mg, clonazepam 2 mg, and zopiclone 7.5 mg at night as required. Actigraphy was undertaken on the 4th day of this admission, which lasted 6 weeks.

**Fig. 1:**
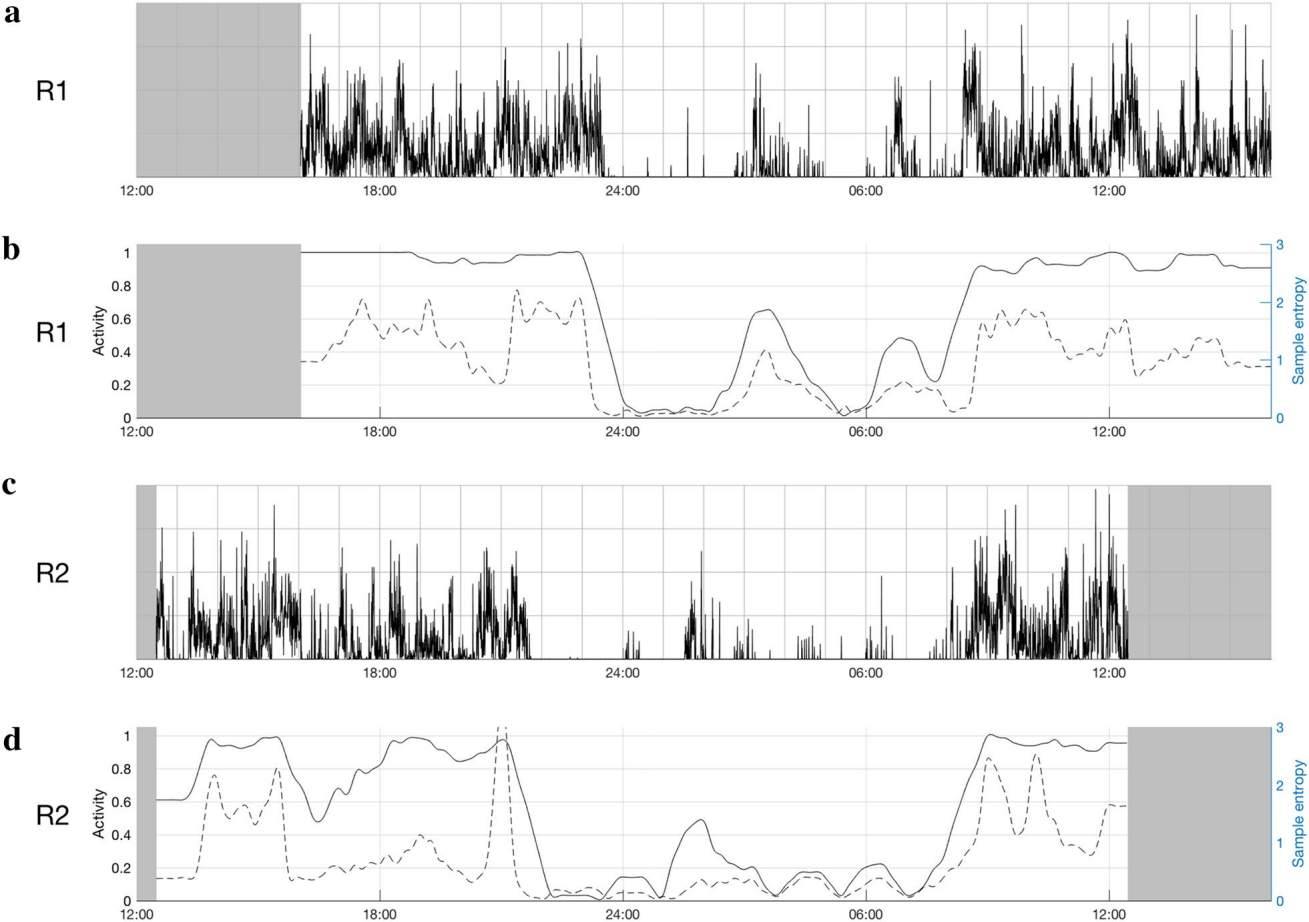
Actigraphy data for two admissions for mania with psychotic symptoms (case 1). **a**, **b** Represent the first recording (R1) and **c**, **d** the second recording (R2). Time of day is shown at the bottom of each chart (24-h clock). Light grey parts at the beginning and end of each chart represent non-monitoring time; **a**, **c** are 24-h actograms: activity counts are shown as black, vertical lines on a scale from 0 to 500 counts. One square in the grid represents 1 h on a horizontal axis and 125 counts on a vertical axis. **b**, **d** The ratio between rest and activity as the fully drawn line (left axis) and sample entropy as the dotted line (right axis)

During the first admission (R1) the patient demonstrated a high mean level of activity over 24 h; the nocturnal sleep interval was short (about 5 h) and disrupted (Fig. 1, panel a). The recording from the second admission (R2) showed a lower 24-h mean activity level characterized by a longer sleep period at night (estimated 7.5 h), lower activity levels and shorter periods of increased activity (panel c). Figure 1 also shows more shifts between rest and activity during the daytime in R2 (panel d), as well as higher peaks of sample entropy compared to R1.

### Case 2—mixed state

Case 2 was aged about 55 years and was hospitalized five times within 3 months due to a fluctuating mixed episode; three hospitalizations included actigraphy monitoring (R1, R2 and R3). The R2 monitoring was undertaken 3 weeks after R1, and R3 was undertaken 8 weeks after R2. R1 began on the third day of admission, and R2 and R3 on the second day of admission. The lifetime history indicated prior experience of psychotic symptoms (auditory hallucinations) and more than 10 hospitalizations in total (including an involuntary hospitalization for severe mania). No important history of alcohol or substance use and no major physical illnesses were reported. The daily medication regime at the actigraphy recordings were valproate 1500 mg, oxazepam 25 mg and quetiapine (R1: 600 mg, R2: 200 mg, R3: 500 mg).

**Fig. 2:**
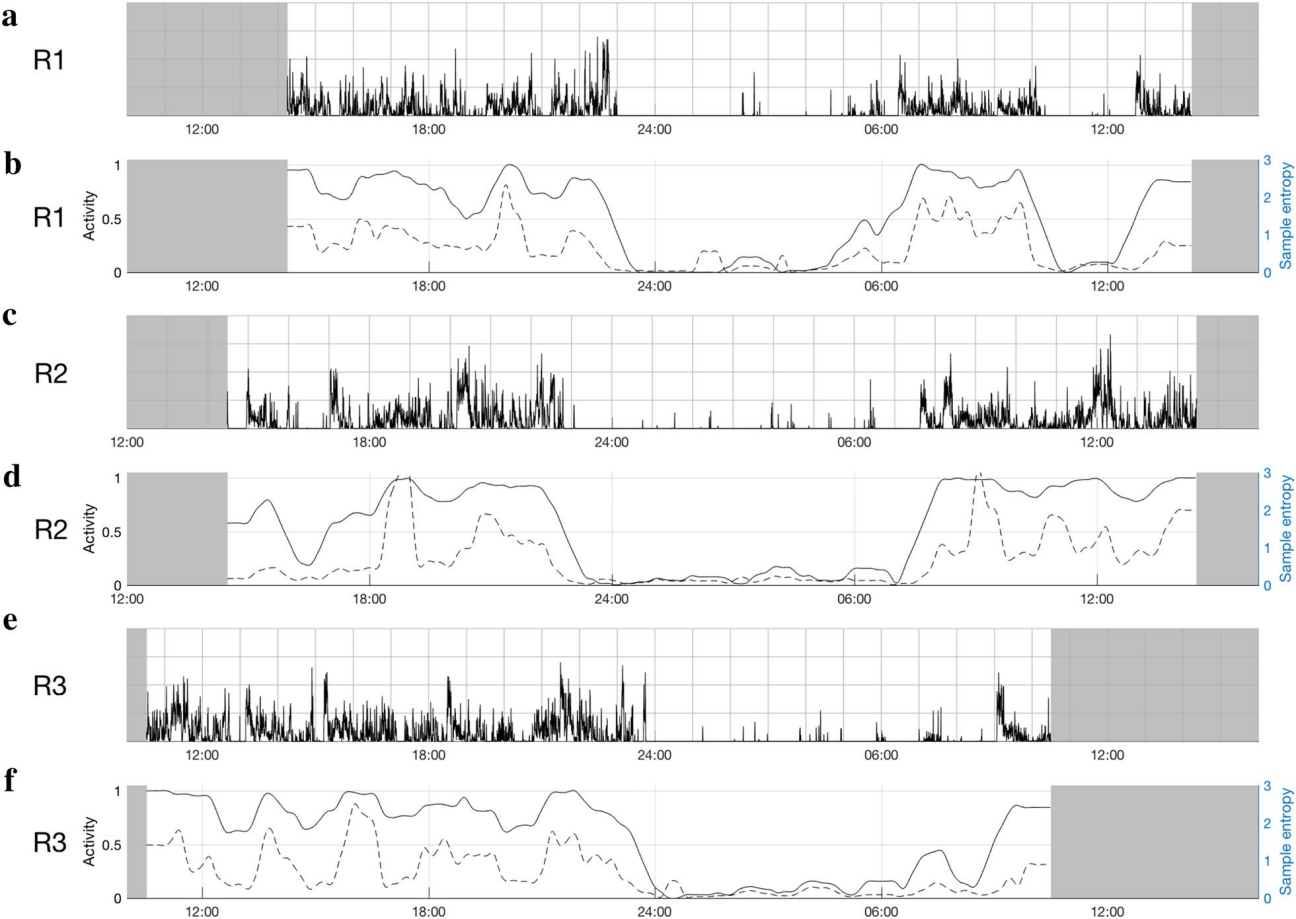
Actigraphy data for three admissions for a mixed state (case 2). **a**, **b** Represent the first recording (R1); **c**, **d** the second recording (R2) and **e**, **f** the third recording (R3). Time of day is shown at the bottom of each chart (24-h clock). Light grey parts at the beginning and end of each chart represent non-monitoring time; **a**, **c**, **e** are 24-h actograms: activity counts are shown as black, vertical lines on a scale from 0 to 500 counts. One square in the grid represents 1 h on a horizontal axis and 125 counts on a vertical axis. **b**, **d**, **f** The ratio between rest and activity as the fully drawn line (left axis) and sample entropy as the dotted line (right axis)

The three actograms in Fig. 2 show consistent patterns across recordings with similar mean levels of activity and similar changes in the distribution of rest and activity periods. The variations in activity parameters may correspond to reported fluctuations in clinical symptoms over 24 h. The patient was experiencing visual hallucinations and paranoid delusions during R2 (Fig. 2, panels c and d), and there is evidence of a greater degree of inactivity during the daytime, with higher peaks of sample entropy (similar to the findings reported in Case 1).

### Case 3—depression and mania

Case 3 was aged between 60 and 65 years. There was evidence of prior alcohol misuse, suicidal ideation and self-harm in the 40-year history of BD. Case 3 had a good response to lithium, but relapses had often followed relatively small modifications to prescribed dosages. The patient was initially admitted in a depressive episode; the daily medication regime at the time of the first actigraphy recording (day 2 of the admission) was quetiapine 275 mg and lithium 210 mg. The admission lasted about 6 weeks until remission. Due to renal complications of lithium, the medication regime was changed to quetiapine and lamotrigine during the admission.

Within 2 weeks, the patient became unwell and was readmitted with a manic episode with intermittent psychotic symptoms. Actigraphy was undertaken at day 2 of the admission; medication at the time of R2 included lamotrigine 200 mg, quetiapine 500 mg, and single doses of oxazepam and alimemazine. The patient improved gradually and was discharged after 10 weeks.

**Fig. 3:**
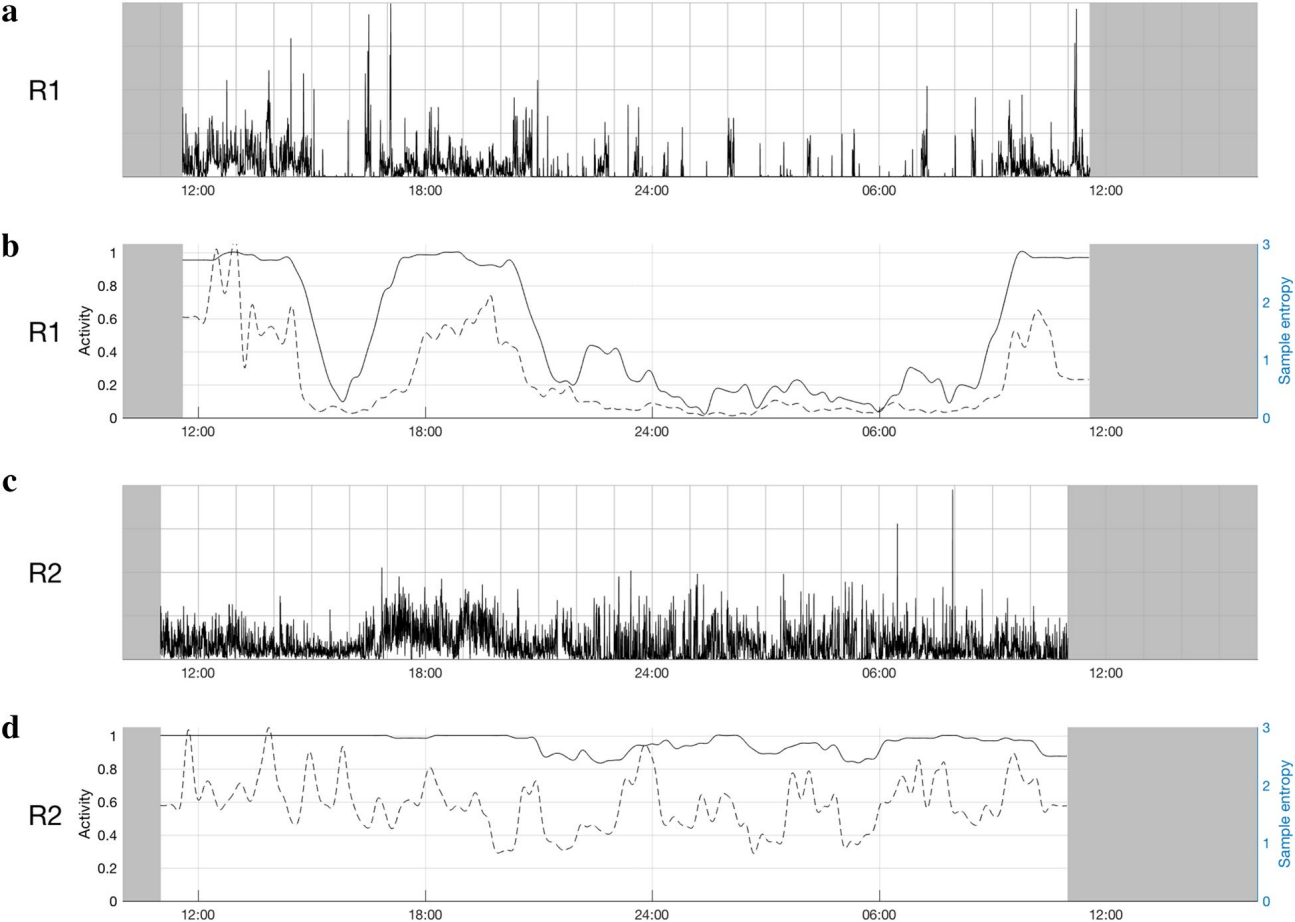
Actigraphy data for two admissions due to depression and mania (case 3). **a**, **b** Represent the first recording (R1) and **c**, **d** the second recording (R2). Time of day is shown at the bottom of each chart (24-h clock). Light grey parts at the beginning and end of each chart represent non-monitoring time; **a**, **c** are 24-h actograms: activity counts are shown as black, vertical lines on a scale from 0 to 500 counts. One square in the grid represents 1 h on a horizontal axis and 125 counts on a vertical axis. **b**, **d** The ratio between rest and activity as the fully drawn line (left axis) and sample entropy as the dotted line (right axis)

As shown in Fig. 3, the activity patterns in acute episodes of depression (R1) and mania (R2) are distinctly different [and, respectively, resemble previous findings (Krane-Gartiser et al. 2014)]. As shown for R1, during depression, periods of motor activity are interspersed with frequent rest periods and the sample entropy is low. The peaks of sample entropy in the afternoon appear to correspond to clinically observed periods of increased anxiety and agitation. During the manic phase, R2 demonstrates an increased total mean level of activity, but also elevated sample entropy (indicating a more complex or irregular activity pattern).

## Conclusions

This case series presents objective recordings of motor activity patterns in three individuals hospitalized with BD episodes and uses the data to indicate the potential of actigraphy combined with mathematical modeling as a within-person diagnostic marker of activity in different acute phases or presentations of BD.

The series of recordings demonstrate that intra-individual activity patterns differ between acute phases of BD, but show within-person consistency for the same phase of illness. For Cases 1 and 2, increasing psychotic symptoms within an episode translated into reduced mean activity levels (case 1), greater variation between rest and activity, and periods with distinctly increased irregularity in activity (sample entropy). Together, these results strengthen the hypothesis that individual movement patterns might be used as an adjunct to basic symptom ratings (e.g., self- or observer-reported mood) and provide a new biological variable for assessment of BD. Of course, to confirm whether this variable is a pure bipolar signal, larger cross-population studies in other psychiatric conditions such as unipolar depression and schizophrenia/schizoaffective disorder should be undertaken. Our data, along with the two recent studies of intra-individual actigraphy in BD (Gershon et al. 2015; Salvatore et al. 2008) indicate that findings from non-linear analysis of actigraphic data could be used to increase diagnostic validity, particularly if the analytical methods are elaborated further (Scott et al. 2016; Salvatore et al. 2008).

Sample entropy (as a measure of pattern irregularity) may be a particularly useful means of characterizing mania, which was also shown in our group study (Krane-Gartiser et al. 2014). With increasing symptom severity within a manic episode in the same individual, it was more complicated to identify robust differences in such a 24-h “snapshot” of activity, but peaks of increased sample entropy and lower energy are indicated as possibilities for further exploration of psychotic symptoms. While the sample entropy and rest/activity ratio appear to display a high degree of covariation in the plots and therefore should be contrasted, it may be that periods when these two measures deviate from each other are of particular interest. Peaks of sample entropy during a stable level of activity could potentially indicate more severe symptoms of psychosis and/or anxiety, as seems to be illustrated by the symptom changes in the three cases (panel d in Fig. 1, panel d in Fig. 2 and panels b and d in Fig. 3). Although the lower mean level of activity might be explained by medication changes (e.g., drugs that increased sedation), sample entropy is less likely to be influenced by pharmacological agents, but has previously been reported in non-manic psychotic patients (Krane-Gartiser et al. 2014; Hauge et al. 2011). Of course, the association between activity patterns and medication should be explored in future studies with larger samples to determine the true significance of irregular movements in psychosis.

This case series has several limitations, primarily related to its exploratory nature in a sample of three BD-I cases. For example, we do not have repeated recordings of hypomania or euthymia, and we cannot take other confounders into account, e.g., some treatments prescribed during the admission may have masked true differences in activity patterns between recordings. Also, we do not have contemporaneous observer ratings or subjective reports of symptoms and thus cannot examine the associations (or direction of causality) between activity and mood changes. Furthermore, each recording was brief (24 h), and recordings over several days would provide more robust data. However, we could not find any published studies reporting non-linear dynamic analyses of repeated within-person recordings undertaken during separate admissions for acute phases of illness. As such, there are no intra-individual data in the literature that support or refute our findings. Long-term studies in larger samples are needed to determine whether real-time objective recordings of rest–activity cycles combined with non-linear analysis are a valuable addition to clinical observation. Actigraphy allows for ecological monitoring and therefore offers the possibility of clarifying the severity and subtype of episode objectively in an individual, which ultimately could be used to offer a biological variable for the assessment of the course and outcome of BD.

## Additional file

**Additional file 1: Table S1.** Clinical data and activity variables for case 1. **Table S2.** Clinical data and activity variables for case 2. **Table S3.** Clinical data and activity variables for case 3.

## Abbreviations

BD: bipolar disorder
BD-I: bipolar disorder, type 1
DSM-5: Diagnostic and Statistical Manual of Mental Disorders, 5th edition
ICD-10: International Classification of Diseases, 10th revision
IRT: item response theory
R1: recording one
R2: recording two
R3: recording three
RMSSD: root mean squared successive difference
SD: standard deviation
