# Supplementary material for: Actigraphy as an objective intra-individual marker of activity patterns in acute-phase bipolar disorder: a case series
Source: Int J Bipolar Disord. 2018 Mar 7;6:8. doi: 10.1186/s40345-017-0115-3 (PMC6161984; doi:10.1186/s40345-017-0115-3)
Supplement: Supplementary file 1 — Additional file 1: Table S1. Clinical data and activity variables for case 1. Table S2. Clinical data and activity variables for case 2. Table S3. Clinical data and activity variables for case 3. [file 40345_2017_115_MOESM1_ESM.docx]

**Additional tables**

**Table S1. Clinical data and activity variables for case 1.**

| **Variable** | **1st admission** | **2nd admission** |
| --- | --- | --- |
| Diagnosis (ICD-10 code) | Mania with psychotic symptoms (F31.2) | Mania with psychotic symptoms (F31.2) |
| Clinical description on the day of recording | Admitted in a manic state with florid psychotic symptoms.  Rapid improvement over 5 days. | Psychotic symptoms were worsening |
| Medication during recording | Olanzapine 5 mg  Oxazepam 20 mg | Quetiapine 250 mg  Clonazepam 2 mg |
| Comments | It is notable that this patient was not on a traditional mood stabilizer. This was a clinical decision based on her long period of stability (about 10 years) without manic episodes. | |
| 24-hour actigraphy recordings | | |
| Mean activity count | 215 | 156 |
| SD in %^1^ | 124.3 | 150.1 |
| RMSSD in %^2^ | 84.0 | 102.4 |
| RMSSD/SD | 0.676 | 0.682 |

^1^SD in %: Standard deviation in percent of mean activity; ^2^RMSSD in %: Root mean squared successive difference in percent of mean activity

**Table S2. Clinical data and activity variables for case 2.**

| **Variable** | **1st admission** | **2nd admission** | **3rd admission** | |
| --- | --- | --- | --- | --- |
| Diagnosis  (ICD-10 code) | Mixed state (F31.6) | Mixed state (F31.6) | Mixed state (F31.6) | |
| Clinical description | Periods of increased motor activity. Noted to have tremor of upper limbs (etiology not recorded). | Changes between being angry and aggressive vs. calm. Paranoid, visual hallucinations, suicidal thoughts. | Changes between aggression and being settled. Neutral mood. | |
| Medication | Quetiapine 600 mg  Valproate 1500 mg  Oxazepam 25 mg | Quetiapine 200 mg  Valproate 1500 mg  Oxazepam 25 mg | Quetiapine 500 mg  Valproate 1500 mg  Oxazepam 25 mg | |
| Comments | There was debate about the differential diagnosis arising from a review of current clinical presentation and previous psychiatric history. However, a multidisciplinary case conference confirmed the diagnosis of a mixed state in a person with BD-I. | | | |
| 24-hour actigraphy recordings | | | | |
| Mean activity count | 83 | 111 | 108 |  |
| SD in %^1^ | 169.1 | 167.3 | 163.3 |  |
| RMSSD in %^2^ | 131.7 | 109.2 | 109.5 |  |
| RMSSD/SD | 0.779 | 0.653 | 0.670 |  |

^1^SD in %: Standard deviation in percent of mean activity; ^2^RMSSD in %: Root mean squared successive difference in percent of mean activity

**Table S3. Clinical data and activity variables for case 3.**

| **Variable** | **1st admission** | **2nd admission** |
| --- | --- | --- |
| Diagnosis (ICD-10 code) | Moderate depression (F31.3) | Mania with psychotic symptoms (F31.2) |
| Clinical description | Worried, depressed mood | Increasingly hectic, verbally and motor active, no sleep |
| Medication during recording | Lithium 210 mg  Quetiapine 275 mg | Lamotrigine 200 mg  Quetiapine 500 mg  Oxazepam (dose unknown)  Alimemazine (stat dose) |
| 24-hour actigraphy recordings | | |
| Mean activity count | 79 | 163 |
| SD in %^1^ | 166.7 | 77.4 |
| RMSSD in %^2^ | 132.3 | 78.3 |
| RMSSD/SD | 0.794 | 1.012 |

^1^SD in %: Standard deviation in percent of mean activity; ^2^RMSSD in %: Root mean squared successive difference in percent of mean activity
